# Severe Manifestation of Hyperparathyroidism With Sagliker Syndrome: A Case Report

**DOI:** 10.7759/cureus.101753

**Published:** 2026-01-17

**Authors:** Mario Manuel Morantes Salazar, Jenny Carolina Salazar Florez, Nathalia Jacome-Pérez, William Darío Arenas Borda, Camilo Andrés Lara Rodríguez

**Affiliations:** 1 General Medicine, Industrial University of Santander, Bucaramanga, COL; 2 Radiology, University of Santander, Bucaramanga, COL; 3 Neuroradiology, Instituto de Diagnóstico Médico, Bucaramanga, COL

**Keywords:** chronic kidney disease-mineral and bone disorder, chronic renal insufficiency, hyperparathyroidism, renal insufficiency, sagliker syndrome

## Abstract

Sagliker syndrome represents a rare and severe manifestation of secondary renal osteodystrophy in patients with end-stage chronic kidney disease. We present the case of a 26-year-old woman on peritoneal dialysis with a one-year history of progressive craniofacial deformity involving both the maxilla and mandible. Laboratory tests demonstrated markedly elevated parathyroid hormone levels (1,936 pg/mL). Cranial CT revealed a diffuse “salt and pepper” pattern, serpiginous lytic trabecular changes, and focal ground-glass areas in the calvarium. Correlation of severe hyperparathyroidism with these characteristic imaging features confirmed Sagliker syndrome. Early recognition is essential to prevent irreversible deformities and long-term morbidity. The patient was managed through a multidisciplinary approach, including medical optimization of secondary hyperparathyroidism, followed by total parathyroidectomy due to persistent biochemical abnormalities and progressive craniofacial deformity.

## Introduction

Secondary hyperparathyroidism is a common complication of advanced chronic kidney disease (CKD), resulting from impaired phosphate excretion, reduced calcitriol synthesis, and chronic hypocalcemia [1]. These metabolic disturbances lead to persistent stimulation of the parathyroid glands, causing excessive secretion of parathyroid hormone (PTH) and sustained high bone turnover. The resulting skeletal remodeling process is collectively referred to as renal osteodystrophy and represents a major component of CKD-mineral and bone disorder [2].

Sagliker syndrome (SS) represents a rare and severe clinical entity that arises as an extreme manifestation of secondary hyperparathyroidism. It is characterized by progressive and often irreversible craniofacial deformities, skeletal expansion, dental abnormalities, and significant functional, psychological, and social impairment. Unlike typical forms of renal osteodystrophy, SS is distinguished by aggressive craniofacial involvement, including marked maxillary and mandibular enlargement, trabecular disruption, cortical expansion, and characteristic radiologic findings [3].

From an epidemiological perspective, SS was first described in 2004 by Sagliker et al. in Turkey in a seminal report that included a small series of patients with severe craniofacial deformities associated with uncontrolled secondary hyperparathyroidism [4]. Subsequent studies have estimated that SS occurs in approximately 0.5% of patients undergoing long-term dialysis, with a marked predominance in young women [5]. To date, fewer than 100 cases have been documented worldwide, most originating from Turkey, followed by reports from Asia and the Middle East. The largest published series was reported by Sagliker et al. in an international multicenter evaluation, which analyzed several dozen patients and further characterized the clinical, biochemical, and genetic features of this syndrome [6]. In Latin America, only isolated cases have been described in countries such as Mexico [7], Ecuador [3], Argentina [8], Brazil, and Colombia [5,9]. The rarity of this condition, together with its clinical overlap with other metabolic bone disorders, contributes to frequent underrecognition and delayed diagnosis. SS is frequently underdiagnosed due to limited awareness of this rare entity, its overlap with other metabolic bone disorders, and delayed recognition of characteristic craniofacial imaging findings in patients with advanced CKD [1,3,4].

The pathophysiology of SS is not fully understood, but it is believed to result from prolonged and uncontrolled secondary hyperparathyroidism leading to an exaggerated bone remodeling response [10]. Chronic elevation of PTH promotes both osteoclastic bone resorption and osteoblastic activity, resulting in disorganized bone formation, trabecular expansion, and skeletal deformity [1,2]. Craniofacial bones appear particularly susceptible due to their high remodeling capacity and prolonged exposure to hormonal imbalance [4]. Importantly, not all patients with severe secondary hyperparathyroidism develop SS, suggesting that biochemical severity alone is insufficient to explain its occurrence [11]. Emerging evidence also supports a genetic predisposition in the development of SS. Cytogenetic abnormalities and mutations involving the *GNAS1* gene, *FGF23*, and *FGFR3* have been reported, suggesting a multifactorial pathophysiological mechanism that extends beyond prolonged biochemical imbalance alone [6,11,12]. These genetic factors may partly explain why only a subset of patients with secondary hyperparathyroidism progress to this catastrophic phenotype.

Beyond skeletal deformities, SS has been associated with multisystem involvement, including neuropsychiatric manifestations, cognitive impairment, hearing loss, speech alterations, behavioral changes, and abnormal electroencephalographic findings, suggesting central nervous system involvement in advanced stages of the disease. These manifestations may significantly impair quality of life and negatively affect treatment adherence, further complicating disease management [13-15].

Given the severity of its clinical consequences and the irreversibility of established skeletal deformities, early identification of SS is critical [16]. Diagnostic imaging plays a pivotal role in recognizing characteristic skeletal patterns, differentiating SS from other metabolic bone diseases, and guiding timely multidisciplinary management. This case represents the third reported case of SS in Colombia and underscores the importance of increasing awareness of this rare but devastating complication of secondary hyperparathyroidism.

## Case presentation

A 26-year-old woman with end-stage CKD (stage 5), previously diagnosed and managed with peritoneal dialysis, was referred for cranial CT due to a one-year history of progressive enlargement of both the maxilla and mandible. At the time of imaging acquisition, the patient provided clinical information as part of a routine diagnostic evaluation, and her medical records were reviewed, documenting a history of arterial hypertension and persistently elevated PTH levels.

Laboratory data obtained from the medical record at the time of imaging demonstrated markedly elevated intact PTH levels (1,936 pg/mL), confirming severe secondary hyperparathyroidism. Additional biochemical abnormalities included mild vitamin D deficiency, hyperphosphatemia, and elevated alkaline phosphatase levels, consistent with impaired calcium-phosphate homeostasis and high bone-turnover activity. The estimated glomerular filtration rate was markedly reduced, in keeping with advanced renal failure requiring peritoneal dialysis. Other laboratory parameters, including serum electrolytes, inflammatory markers, complete blood count, and liver function tests, were within normal limits, with no evidence of concomitant infectious, inflammatory, or systemic disease. Full laboratory results are summarized in Table 1.

**Table 1:** Initial laboratory findings of the patient. PTH: parathyroid hormone; eGFR: estimated glomerular filtration rate

Parameter	Patient’s value	Reference range
Intact PTH	1,936 pg/mL	10–65 pg/mL
Total calcium	9.2 mg/dL	8.4–10.2 mg/dL
Calcium (albumin-corrected)	8.8 mg/dL	8.4–10.2 mg/dL
Phosphorus	6.5 mg/dL	2.5–4.5 mg/dL
Alkaline phosphatase	500 U/L	44–147 U/L
25-Hydroxyvitamin D	12 ng/mL	20–50 ng/mL
Ionized calcium	1.00 mmol/L	1.12–1.32 mmol/L
Albumin	3.4 g/dL	3.5–5.0 g/dL
Creatinine	6.2 mg/dL	0.7–1.2 mg/dL
eGFR (estimated)	9 mL/minute/1.73 m²	>90 mL/minute/1.73 m²

**Figure 1:**
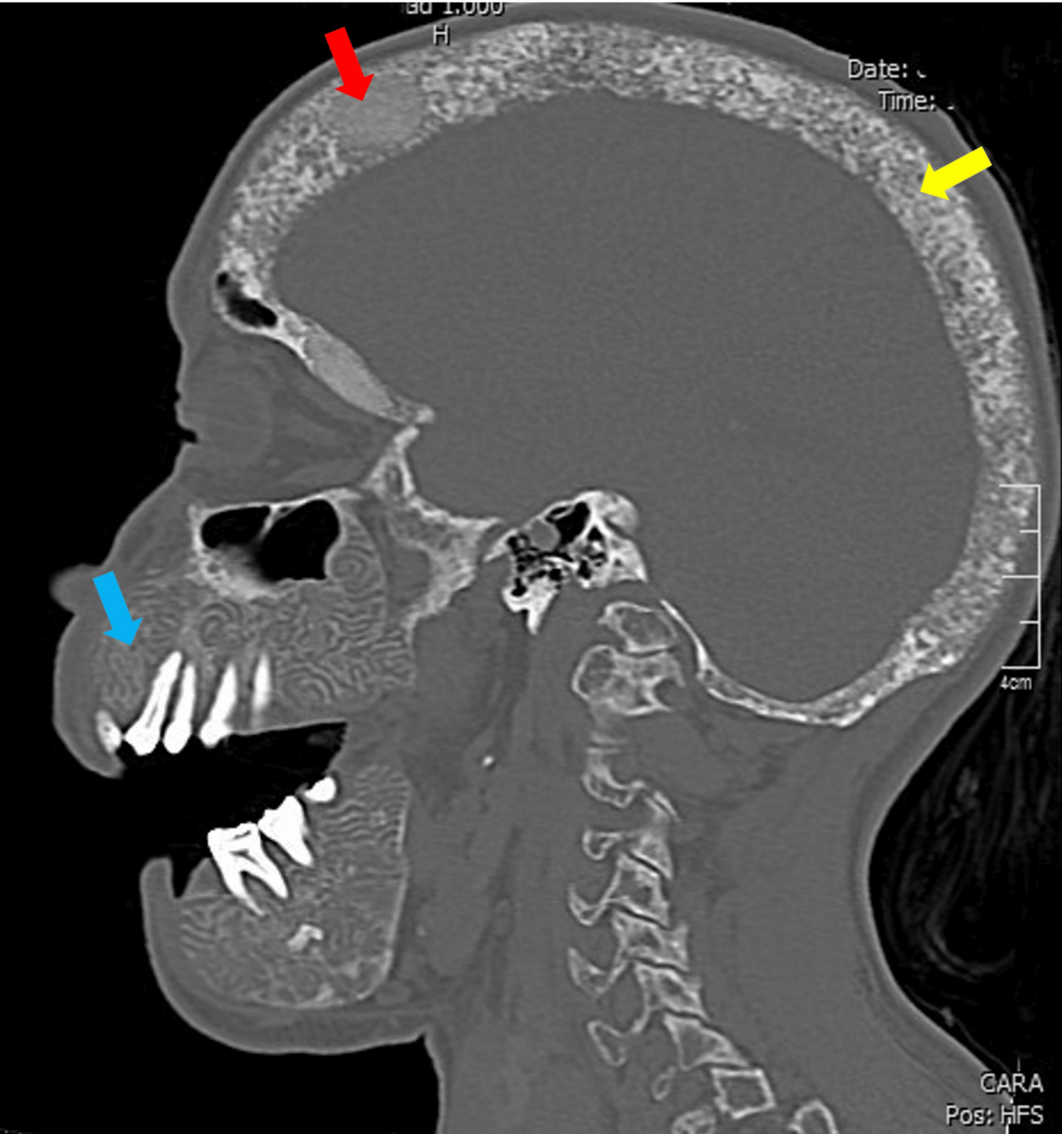
CT of the skull in bone window. Sagittal reconstruction showing diffuse craniofacial bone thickening and deformity, with marked maxillary and mandibular overgrowth with significant cortical expansion (blue arrow), diffuse “salt and pepper” appearance predominantly in the calvaria and skull base (yellow arrow), and rounded focal ground-glass regions within the calvarium (red arrow).

**Figure 2:**
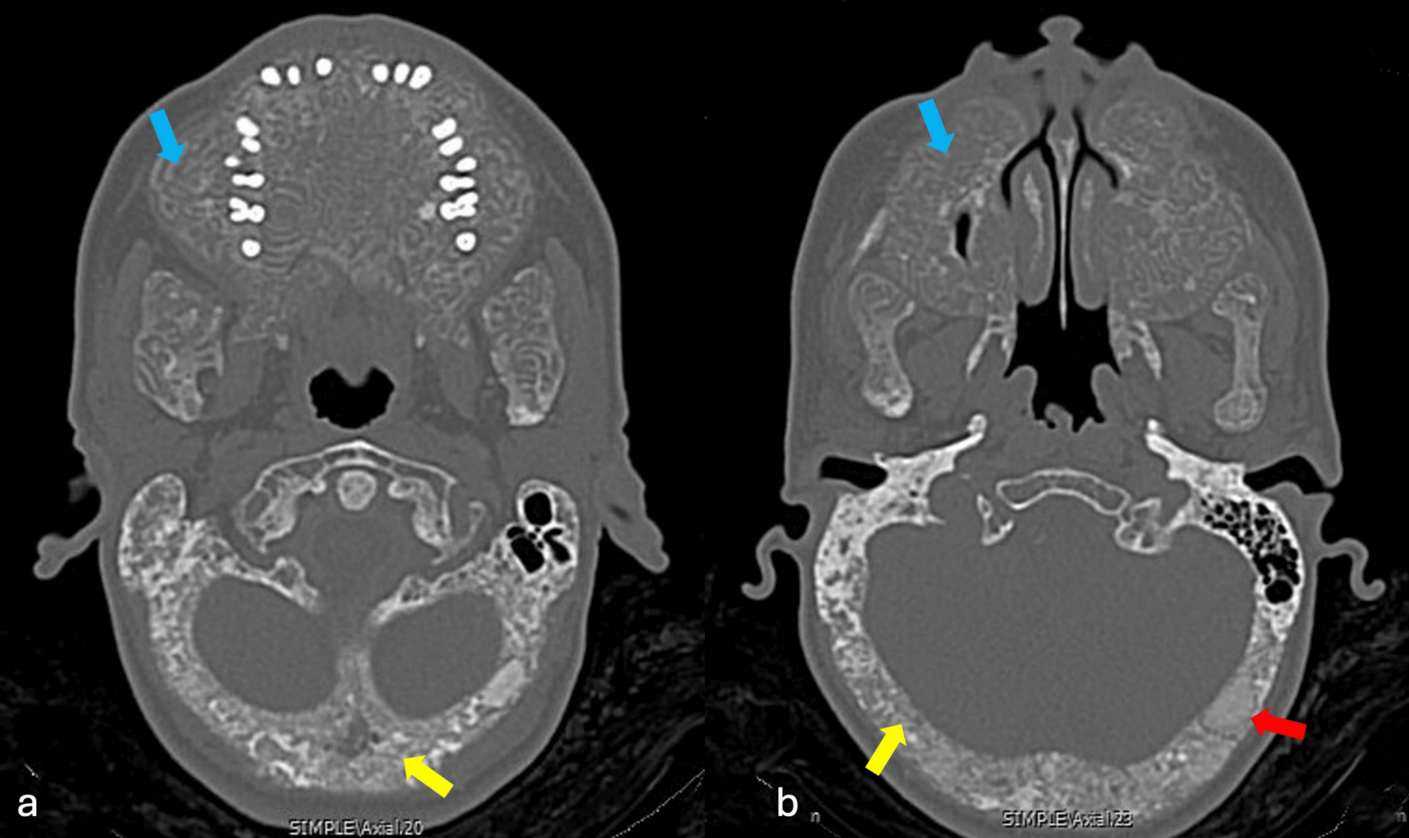
CT of the skull in bone window axial images. (A, B) Diffuse craniofacial bone thickening and deformity, with marked maxillary and mandibular overgrowth with significant cortical expansion (blue arrow), diffuse “salt and pepper” appearance predominantly in the calvaria and skull base (yellow arrow), and rounded focal ground-glass regions within the calvarium (red arrow).

Clinically, the patient denied neurosensory deficits, orofacial pain, dysphagia, dyspnea, weight loss, fever, or other constitutional symptoms at the time of evaluation. Non-contrast cranial CT demonstrated a diffuse salt-and-pepper appearance predominantly affecting the calvaria and skull base, along with rounded ground-glass regions within the calvarium. In addition, there was heterogeneous trabecular disruption with serpiginous lytic changes and marked cortical expansion involving both the maxilla and mandible. These findings were highly suggestive of severe secondary renal osteodystrophy consistent with an SS phenotype (Figures 1, 2).

Subsequent clinical information regarding disease management and evolution was obtained through a telephone follow-up with the patient. According to her medical history and self-reported information, she underwent multidisciplinary evaluation, and treatment was initiated with dietary phosphate restriction, phosphate binders, vitamin D analog supplementation, and optimization of peritoneal dialysis. Due to persistently elevated PTH levels and progressive craniofacial deformity, surgical management with total parathyroidectomy was performed. Postoperative follow-up reportedly demonstrated biochemical stabilization. Further longitudinal clinical follow-up and imaging surveillance were not available; therefore, progression or stabilization of craniofacial deformities after surgery could not be assessed.

## Discussion

SS represents the most severe complication of secondary hyperparathyroidism and the most exaggerated and irreversible form of renal osteodystrophy in patients with end-stage CKD. In 2004, Sagliker et al. first reported a case series from Turkey [4], with a higher incidence in women between 18 and 39 years old. However, not all patients with hyperparathyroidism and CKD develop SS, and its exact pathophysiology remains unclear [5,8].

Proposed contributing factors include loss of renal function with decreased synthesis of calcitriol (active vitamin D) and reduced renal reabsorption, leading to chronic hypocalcemia and continuous stimulation of PTH [13,15]. This results in markedly increased bone turnover, deformities, and brown tumors [14]. In addition, a potentiating effect from vitamin C deficiency, resulting from malnutrition and loss of water-soluble vitamins during hemodialysis, has been suggested [13]. Possible genetic susceptibility (*FGF23*, *GNAS1*) has also been proposed as a factor promoting SS development [6].

Our case describes a 26-year-old female patient with craniofacial bone abnormalities characteristic of SS and markedly elevated PTH levels. These findings are consistent with previously reported international cases describing progressive craniofacial deformity with imaging findings such as the “salt and pepper” pattern, serpiginous lytic lesions, and ground-glass opacities, considered distinctive radiological features of SS [1,2,4,7,11]. Additional findings reported in the literature include maxillary and mandibular protrusion, dental extrusion, intraoral soft-tissue hyperplasia, vertebral compression, and nasal bone destruction [7,10,13,16]. Maxillary protrusion was identified in our patient; however, no further studies were performed to assess vertebral involvement. In their comparative cephalometric analysis with patients with SS, Mi et al. found significant differences in linear measurements (lower anterior facial height and total facial height) with Class II malocclusion, findings that could help differentiate patients with SS from those with hyperparathyroidism alone [16]. Although these linear measurements were not performed in our patient, it would be useful to apply them in future cases with similar diagnostic suspicion.

Additionally, it has been reported that patients with SS may present with expansive bone lesions consistent with brown tumors in long bones such as the humerus, femur, and phalanges, causing pain, deformity, and functional limitations [11,16]. In our patient, no additional imaging studies were available to determine the presence of further head and skull involvement. SS can also be associated with pain, functional limitations, hearing loss, neurological impairment, and severe emotional and psychiatric symptoms such as depression or anxiety, leading to a significant decline in quality of life and poor adherence to treatment [4,14,16]. In this case, the patient had not yet developed neurosensory or cognitive impairment, representing a potential therapeutic window of opportunity; however, longitudinal clinical and imaging follow-up was unavailable, preventing the evaluation of subsequent neurological or functional progression.

Beyond skeletal abnormalities, SS has been associated with multisystem manifestations including pain, functional limitation, hearing loss, neurological deterioration, and severe emotional or psychiatric symptoms such as depression and anxiety, all of which may significantly impair quality of life and treatment adherence [12,14]. In the present case, the patient had not yet developed neurosensory deficits, hearing impairment, or cognitive deterioration, representing a potential therapeutic window of opportunity. However, longitudinal clinical and imaging follow-up was not available, precluding assessment of subsequent neurological or functional progression.

Although established skeletal deformities are irreversible [10], this suggests that early surgical parathyroidectomy can halt disease progression if performed before severe architectural distortion occurs [17]. Likewise, Panezai et al. in the United States demonstrated that even with adequate dialysis, SS may progress if hyperparathyroidism is not effectively corrected pharmacologically or surgically [18]. Therefore, optimal management requires a multidisciplinary approach including nephrology, endocrinology, radiology, and maxillofacial surgery [17]. Control of secondary hyperparathyroidism is essential, and surgical parathyroidectomy remains the only intervention capable of stabilizing skeletal progression.

From a radiologic perspective, the differential diagnosis of SS includes fibrous dysplasia, Paget’s disease, isolated brown tumors related to secondary hyperparathyroidism, and metabolic bone disease due to vitamin D deficiency [19]. Fibrous dysplasia typically demonstrates a homogeneous ground-glass matrix with bone expansion but lacks the diffuse salt-and-pepper pattern and severe biochemical abnormalities characteristic of SS [2,20]. Paget’s disease more commonly affects older patients and is characterized by cortical thickening, coarsened trabeculae, and mixed lytic-sclerotic changes rather than diffuse trabecular resorption [2]. Brown tumors associated with secondary hyperparathyroidism may appear as focal lytic lesions but do not usually produce the extensive craniofacial expansion seen in SS [16,19]. Vitamin D-related metabolic bone disease generally presents with osteopenia and insufficiency fractures without aggressive craniofacial remodeling [15,19,20]. Accurate differentiation relies on integration of imaging findings with clinical context and laboratory data, highlighting the critical role of radiologists in early diagnosis [1,2]. Table 2 summarizes the main imaging characteristics of SS and its principal radiologic differential diagnoses.

**Table 2:** Main imaging characteristics of Sagliker syndrome and its principal radiologic differential diagnoses. Characteristic imaging findings of Sagliker syndrome with those of its main differential diagnoses, including fibrous dysplasia, Paget’s disease, brown tumors associated with secondary hyperparathyroidism, and metabolic bone disease related to vitamin D deficiency. CKD: chronic kidney disease; PTH: parathyroid hormone; SHPT: secondary hyperparathyroidism

Condition	Typical age group	Key imaging features	Laboratory findings	Distinguishing features
Sagliker syndrome	Young adults with CKD	Diffuse “salt and pepper” skull, serpiginous lytic changes, cortical expansion, ground-glass areas	Markedly elevated PTH, hyperphosphatemia	Aggressive craniofacial expansion, systemic involvement
Fibrous dysplasia	Children and young adults	Homogeneous ground-glass matrix, bone expansion	Usually normal	No severe biochemical abnormalities, focal involvement
Paget’s disease	Older adults	Cortical thickening, coarsened trabeculae, mixed lytic-sclerotic pattern, metaphyseal osteolytic appearance, bone enlargement, and reticular bone	Elevated alkaline phosphatase	Age-related, rarely severe facial deformity
Brown tumors (SHPT)	CKD patients over 50 years old	unifocal or multifocal osteolytic bone lesions, well-defined, in some with progressive growth	Elevated PTH	Localized lesions without diffuse craniofacial expansion
Vitamin D deficiency	Any age	Osteopenia, looser zones	Low vitamin D, normal/mild PTH	No aggressive bone remodeling

**Figure 3:**
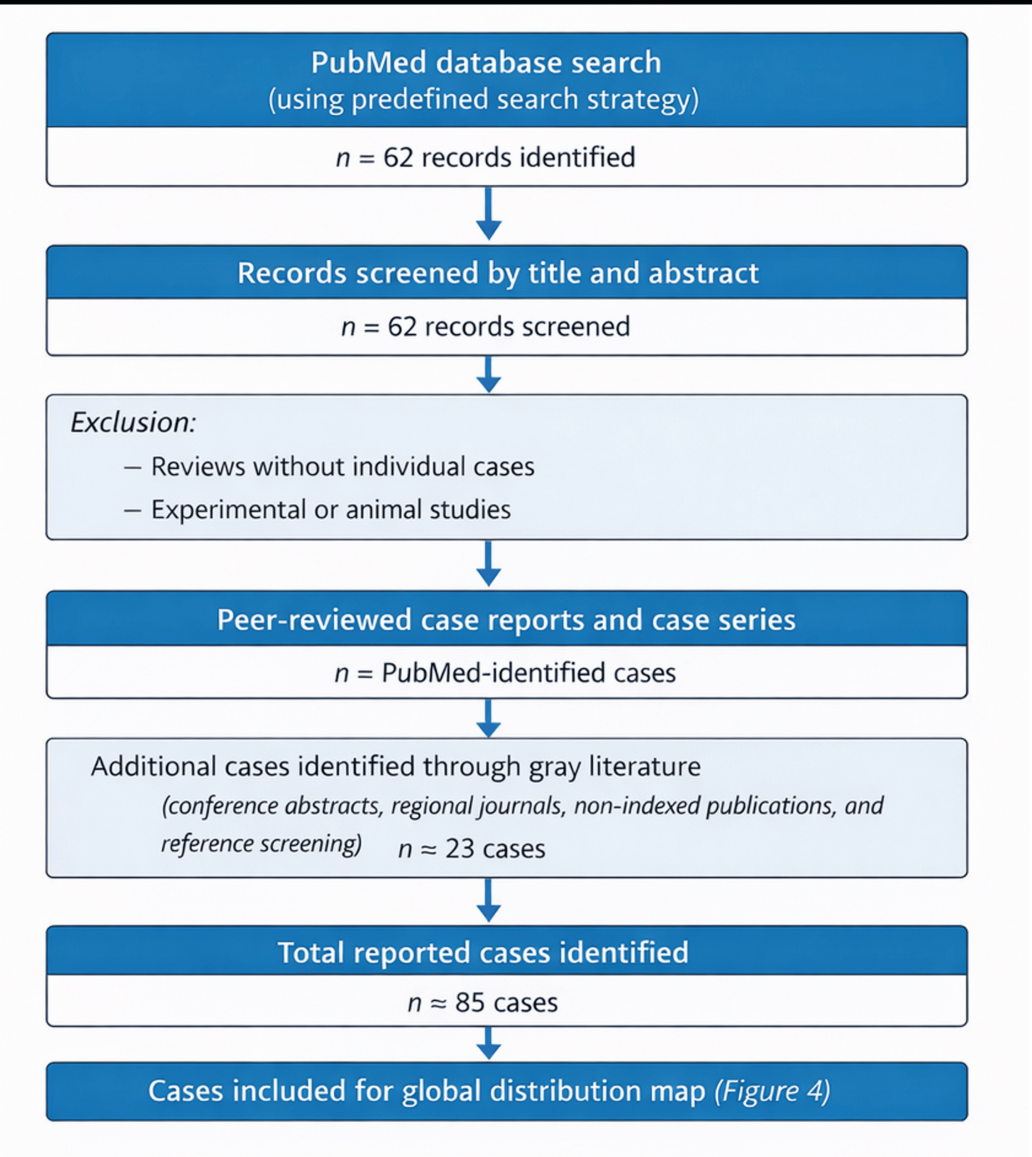
Literature search and case identification process for Sagliker syndrome. This flow diagram illustrates the literature search strategy used to identify reported cases of Sagliker syndrome. A broad search of the PubMed database identified 62 relevant records using predefined search terms. Additional cases were identified through gray literature sources, including non-indexed journals, regional publications, conference abstracts, and reference screening. Search terms: (“Sagliker syndrome”[All Fields] OR “Sagliker”[All Fields] OR “uremic leontiasis ossea”[All Fields] OR “leontiasis ossea”[All Fields]) AND (“hyperparathyroidism”[All Fields] OR “secondary hyperparathyroidism”[All Fields] OR “chronic kidney disease”[All Fields] OR “end stage renal disease”[All Fields] OR “renal osteodystrophy”[All Fields]) Filters: Abstract.

**Figure 4:**
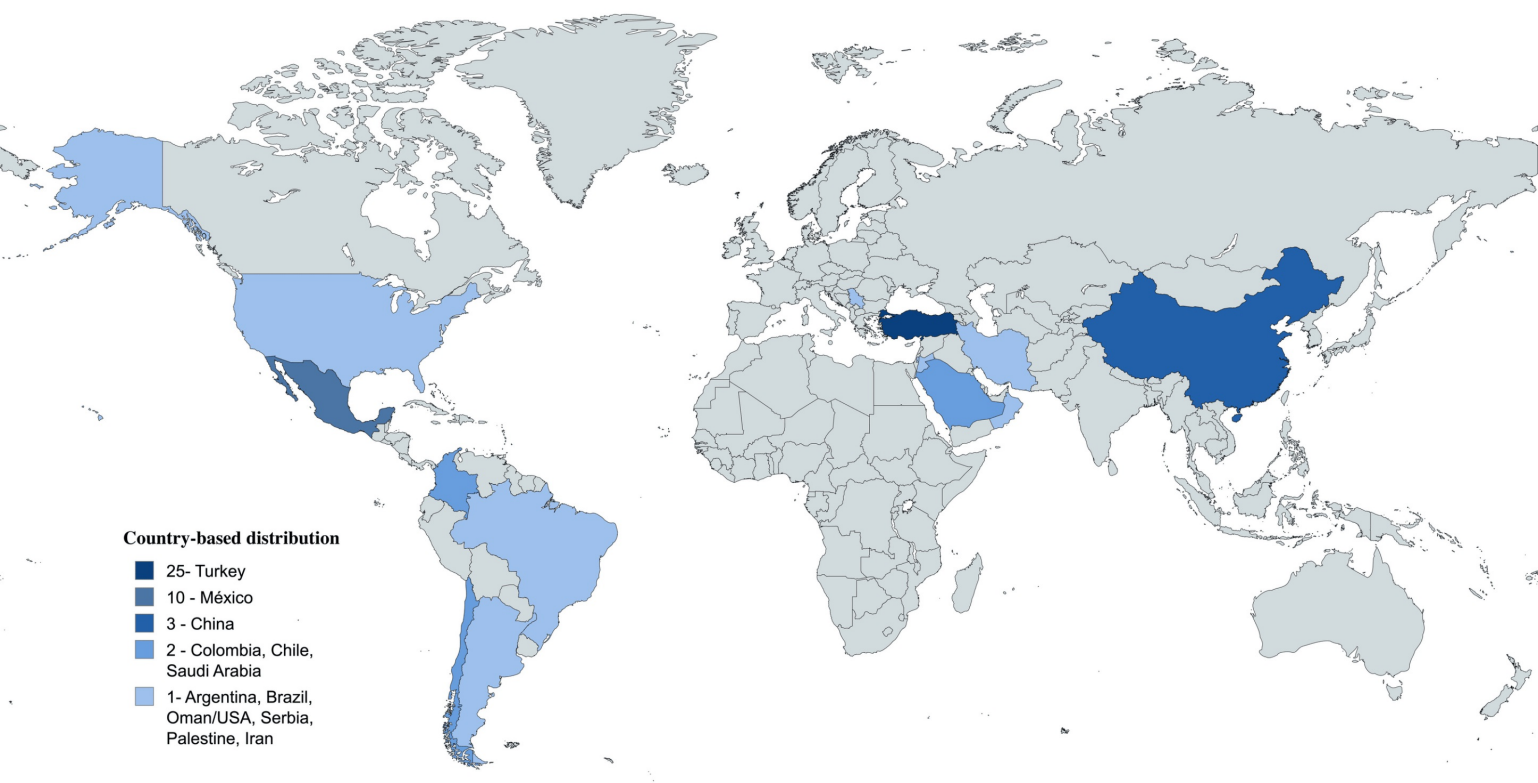
Global distribution of reported Sagliker syndrome cases. The figure was created entirely by the authors using the MapChart platform (https://www.mapchart.net/) based on data extracted from published case reports and case series. The map illustrates the number of cases reported by country, with darker shades representing higher frequencies. Turkey shows the largest number of reported cases (n = 25), followed by Mexico (n = 10) and China (n = 3). Fewer cases have been described from Colombia, Chile, and Saudi Arabia (n = 2 each), and isolated reports exist from Argentina, Brazil, the United States, Oman, Palestine, Serbia, and Iran (n = 1 each). This geographic distribution underscores the rarity of the syndrome and highlights the need to maintain a high index of diagnostic suspicion in young patients with chronic kidney disease and secondary hyperparathyroidism. Note: This visualization is intended for descriptive and educational purposes only; it does not reflect epidemiologic prevalence or incidence.

A literature review demonstrates that SS and uremic leontiasis ossea represent rare entities, with approximately 85 cases reported worldwide to date. This estimate is based on a broad literature search conducted in PubMed, which identified 62 relevant articles using predefined search terms, complemented by additional cases identified through gray literature sources, including non-indexed journals, regional publications, conference abstracts, and reference screening (Figure 3). Most reported cases originate from Turkey, including the original case series, as well as a large multinational series comprising patients from Asia and the Middle East [4,6]. In Latin America, only isolated cases have been described, with Mexico reporting the largest number of cases, while Chile, Argentina, Brazil, and Ecuador have sporadic reports [3,7,8]. In Colombia, two cases have been previously documented [5,9]; therefore, the present report represents the third published case in the country. Figure 4 illustrates a map summarizing the geographic distribution of reported cases, highlighting the rarity of this condition and underscoring the importance of maintaining a high index of suspicion in young patients with CKD and secondary hyperparathyroidism. The true prevalence of SS e is likely underestimated, as unpublished cases and reports in non-indexed or non-English journals may not be captured by conventional database searches.

This report has several limitations inherent to its case-based design. First, longitudinal clinical, laboratory, and imaging follow-up data were not available, which precluded assessment of disease progression or stabilization after surgical treatment. Second, the findings are based on a single patient, limiting generalizability to broader populations. Finally, pre-referral biochemical trends and earlier imaging studies were unavailable, restricting evaluation of the temporal evolution of secondary hyperparathyroidism and its skeletal manifestations. Despite these limitations, this case provides valuable educational insight into the radiologic recognition and clinical relevance of SS.

## Conclusions

SS represents a rare and severe manifestation of secondary hyperparathyroidism in patients with CKD, characterized by marked maxillofacial deformities and profound impact on quality of life. Diagnostic imaging plays a crucial role by enabling early recognition, characterization, and differentiation from other bone disorders, which is essential to guide appropriate management. This case, the third reported from Colombia, highlights the importance of increasing awareness and promoting further research of this entity to optimize diagnosis and deepen global understanding.
